# UV-B Radiation Largely Promoted the Transformation of Primary Metabolites to Phenols in *Astragalus mongholicus* Seedlings

**DOI:** 10.3390/biom10040504

**Published:** 2020-03-26

**Authors:** Yang Liu, Jia Liu, Ann Abozeid, Ke-Xin Wu, Xiao-Rui Guo, Li-Qiang Mu, Zhong-Hua Tang

**Affiliations:** 1 School of Forestry, Northeast Forestry University, Harbin 150040, China; 2 Material Science and Engineering College, Northeast Forestry University, Harbin 150040, China; 3 Key Laboratory of Plant Ecology, Northeast Forestry University, Harbin 150040, China; 4 Botany Department, Faculty of Science, Menoufia University, Shebin El-koom 32511, Egypt

**Keywords:** *Astragalus mongholicus*, ultraviolet-B radiation, phenolics, untargeted gas chromatography-mass spectrometry, targeted liquid chromatography-mass spectrometry

## Abstract

Ultraviolet-B (UV-B) radiation (280–320 nm) may induce photobiological stress in plants, activate the plant defense system, and induce changes of metabolites. In our previous work, we found that between the two *Astragalus* varieties prescribed by the Chinese Pharmacopoeia, *Astragalus mongholicus* has better tolerance to UV-B. Thus, it is necessary to study the metabolic strategy of *Astragalus* under UV-B radiation further. In the present study, we used untargeted gas chromatography-mass spectrometry (GC-MS) and targeted liquid chromatography-mass spectrometry (LC-MS techniques) to investigate the profiles of primary and secondary metabolic. The profiles revealed the metabolic response of *Astragalus* to UV-B radiation. We then used real-time polymerase chain reaction (RT-PCR) to obtain the transcription level of relevant genes under UV-B radiation (UV-B supplemented in the field, λ_max_ = 313 nm, 30 W, lamp-leaf distance = 60 cm, 40 min·day^−1^), which annotated the responsive mechanism of phenolic metabolism in roots. Our results indicated that supplemental UV-B radiation induced a stronger shift from carbon assimilation to carbon accumulation. The flux through the phenylpropanoids pathway increased due to the mobilization of carbon reserves. The response of metabolism was observed to be significantly tissue-specific upon the UV-B radiation treatment. Among phenolic compounds, C6C1 carbon compounds (phenolic acids in leaves) and C6C3C6 carbon compounds (flavones in leaves and isoflavones in roots) increased at the expense of C6C3 carbon compounds. Verification experiments show that the response of phenolics in roots to UV-B is activated by upregulation of relevant genes rather than phenylalanine. Overall, this study reveals the tissues-specific alteration and mechanism of primary and secondary metabolic strategy in response to UV-B radiation.

## 1. Introduction

*Astragalus mongholicus* (*A. mongholicus*) is an important perennial herb of the Legumes family [1,2]. Its dried roots (Radix astragali) are one of the most popular Chinese herbal medicines in East Asia and are considered as healthy food in Western countries [3,4]. Radix astragali is often used as an antiperspirant, a vimmuno-stimulant, and a supplementary medicine during cancer therapy [5,6,7]. Calycosin-7-*O*-β-d-glucoside (CAG) can potentially be used as a “marker compound” for the chemical evaluation or product standardization of Radix astragali [8,9,10].

The need for *A. mongholicus* to capture sunlight for photosynthesis inevitably exposes the plant to ultraviolet (UV) radiation. Ultraviolet radiation is the general term for three radiation wavelengths: UV-C (200–280 nm), UV-B (280–320 nm), and UV-A (320–400 nm). Ultraviolet-C is completely absorbed by atmospheric gases. Ultraviolet-Ais barely absorbed by atmospheric ozone, but UV-A impact on plants is small. Ultraviolet-B is potentially harmful to plants but is partially absorbed by ozone [11,12,13]. It was reported that the influx of UV-B radiation will likely increase as a result of the depletion of stratospheric ozone [14]. Increased amounts of UV-B radiation affect plant development, morphology, and physiology [15]. The sessile lifestyle of plants forces them to adapt to dynamic environmental conditions. To counteract these problems, plants use a range of strategies, including increases in leaf thickness, UV-B reflective properties, and the accumulation of UV-B-absorbing secondary metabolites. The most common protective mechanism against potentially damaging irradiation is the biosynthesis of UV-absorbing compounds [16]. These secondary metabolites mostly consist of phenolic compounds, flavonoids, and hydroxycinnamate esters, which accumulate in the vacuoles of epidermal cells in response to UV-B radiation and attenuate the penetration of the UV-B portion of the solar spectrum into deeper cell layers with little effect on the visible region. These responses to UV-B radiation may result in the reprogramming of metabolites in *A. mongholicus* and even altered accumulation of bioactive compounds. Given this fact, it is essential to better understand the adaptive responses of metabolites in *A. mongholicus* to increased UV-B radiation.

Phenolic compounds play diverse roles in plants. These compounds provide structural support of the cell wall or protect plants against pathogens, herbivores, and UV radiation [17]. Of all classes of secondary metabolites, phenolics, specifically flavonoids, are the most relevant for UV protection. Plant phenolics are compounds having at least one aromatic ring substituted with at least one hydroxyl group. The hydroxyl group can be free or engaged in another function as an ether, ester, or glycoside [18,19,20]. Phenolics exhibit a large variety of structures in nature and can be divided into three groups according to their chemical structure: (1) compounds having a C6C1 carbon skeleton, such as 4-hydroxybenzoic acid, vanillic acid, and salicylic acid; (2) compounds having a C6C3 carbon skeleton, such as caffeic acid, *p*-coumaric acid, and ferulic acid; and (3) compounds having a C6C3C6 carbon skeleton (flavonoids are typical C6C3C6 phenolic compounds) [21,22]. Phenolic compounds are generally synthesized via the shikimate pathway. The shikimate pathway is a major biosynthetic route for both primary and secondary metabolism. This pathway begins with phosphoenolpyruvate and erythrose-4-phosphate and ends with chorismate [23,24]. Phenylalanine, a key metabolite, is synthesized by chorismate [25]. Phenylalanine is considered the general precursor of C6C1-, C6C3-, and C6C3C6 compounds and their polymers in plant metabolism [26]. Many investigations have closely focused on the accumulation of phenolic compounds, which is regulated by biotic and abiotic stress [27]. *Astragalus mongholicus* is considered a rich source of natural phenolic compounds [28,29].

Regarding the complex primary and secondary metabolites fluctuations of higher plants, metabolomics plays a vital role as a suitable tool [15,30]. Metabolomics approaches are becoming more widely used in modern biology [28,31,32]. In plant metabolomics studies, common analytical technologies include liquid chromatography-mass spectrometry (LC-MS), gas chromatography-mass spectrometry (GC-MS), and nuclear magnetic resonance spectroscopy (NMR), among others [33,34,35]. Gas chromatography-mass spectrometry is widely used in metabolomic studies due to its high quality and reproducibility, wide dynamic range, universal mass spectral library and ability to detect hydrophilic metabolites after derivatization [36]. A reference pool of GC-MS for many primary metabolites, such as sugars, organic acids, fatty acids, and amino acids, has been established [37,38,39]. The short run time and relatively low running cost are strong advantages of GC-MS [40]. In contrast, LC-MS can potentially analyze a wide variety of large hydrophobic metabolites predominant in secondary compounds in plants; these compounds include phenolic compounds, terpenoids and alkaloids [41,42,43]. Moreover, the development of the ultra-performance liquid chromatography-mass spectrometry (UPLC-MS) methods leads to more powerful analyses of metabolites because of its higher throughput and shorter run time than those of conventional HPLC-MS [44,45]. Accordingly, the combination of GC-MS and LC-MS could clearly enhance the coverage of metabolites, allowing both a comprehensive overview and detailed analysis of metabolic changes in plants [46]. Recently, these two methods have been jointly adopted for many botanical studies [30,47,48,49].

Many of the biosynthetic enzymes of phenolics, such as phenylalanine ammonia lyase (*PAL*, E.C. 4.3.1.25) and chalcone synthase (*CHS*, E.C. 2.3.1.74), are activated by UV-B [1,50]. Flavonoid biosynthesis and their regulation have been thoroughly investigated. As the major bioactive compound, the calycosin 7-*O*-glucoside (CAG) biosynthesis pathway has been completely elucidated based on its presence in other legumes [10,51,52,53]. Calycosin 7-*O*-glucoside is synthesized from L-phenylalanine via the isoflavonoid branch of phenylpropanoid metabolism. *PAL*, cinnamate 4-hydroxylase (*C4H*, E.C. 1.14.13.11) and 4-coumarate-coenzyme A ligase (*4CL*, E.C. 6.2.1.12) are enzymes involved in the upstream general phenylalanine pathway. Isoliquiritigenin, an isoflavonoid skeleton, is synthesized via *CHS* and chalcone reductase (*CHR*), co-catalyzed by the condensation of 4-coumaroyl-CoA and three molecules of malonyl-CoA. Afterward, a series of chemical reactions is performed under the catalysis of chalcone isomerase (*CHI*, E.C. 5.5.1.6), isoflavone synthase (*IFS*, E.C. 1.14.13.86), isoflavone *O*-methyltransferase (*IOMT*) and isoflavone 3′-hydroxylase (*I3′H*). At the last step, the formation of CAG from calycosin (CA) is catalyzed by uridine diphosphate glucose (UDP)-glucose: calycosin 7-O-glucosyltransferase (*UCGT*). For the last step, the formation of CAG from CAis catalyzed by uridine diphosphate glucose (UDP)-glucose: calycosin 7-*O*-glucosyltransferase (*UCGT*) [54,55].

Due to the release of anthropogenic pollutants such as chlorofluorocarbons, a larger proportion of the UV-B spectrum reaches the surface of the earth and affects all living organisms [56,57]. In general, the most common protective mechanism against potentially damaging irradiation in plants is the biosynthesis of UV-absorbing compounds, such as phenolic compounds, flavonoids, and hydroxycinnamate esters [16]. The use of UV-B radiation is expected to induce the accumulation of CAG in *A. mongholicus* [55,58]. This could satisfy consumer demand for these naturally derived health-promoting products. Our goal is to understand the response of the metabolites in *A. mongholicus* to UV-B radiation. To the best of our knowledge, no information on the metabolites response mechanism of *A. mongholicus* to UV-B radiation is currently available. To deeply explore the response and mechanism explaining how metabolic reprogramming can achieve a new steady state under increased UV-B radiation, we investigated the influence on the specific tissue accumulation of primary and phenolic metabolites using untargeted (GC-MS) and targeted (LC-MS) metabolomics in *A. mongholicus* under increased UV-B radiation.

## 2. Materials and Methods

### 2.1. Plant Materials, Growth Conditions and Treatments

Seeds of *A. mongholicus* were sown in Botanical Garden of the Key Laboratory of Plant Ecology, Northeast Forestry University, Harbin, northeastern of China (natural environment at east longitude 126°38′, north latitude 45°43′). All plants grew under standard field conditions, with an average temperature of 16 °C during the day (14 h) and 4 °C at night (10 h), and water was applied every two days. After three months, plants of uniform height and stem thickness were transferred to containers (37 cm × 28 cm × 10 cm) and used for this study. All containers (*n* = 6) contained equal bulk density of potting mix and 12 plants, half of the containers for UV-B treated plants and the other for controlled plants. Plants were treated in the field with UV irradiation (λ_max_ = 313 nm, 30 W, lamp-leaf distance = 60 cm, 40 min·day^−1^). The UV-B irradiation intensity (33.5 μW/cm^2^) was monitored by a UV light meter (UV340B, Xin Bao Technologies, ShenZhen, China). That is, the irradiation doses of UV-B treated group was 804 J m^−2^, the dose chosen was based on our previous study [57]. Control plants were maintained in the previous environment. Sampling was performed for both plants treated with UV and controlled plants at 10 days after treatment. Morphological indicators (plant height, fresh weight of root and whole plant, leaf area) were recorded for 20 biologic repetitions, firstly. Then, these plants were sampled. Collected plants were rinsed briefly in deionized water and separated into roots, stems, leaves and petioles. The samples were then immediately frozen in liquid nitrogen and stored at −80 °C for biochemical analysis. Six biological replicates were prepared for each sample. The analysis of H_2_O_2_ and antioxidant enzymes was performed based on the methods of Liu et al. [59,60,61]. Chlorophyll analysis was performed in accordance with the methods of Arnon [62,63].

### 2.2. Primary Metabolite Extraction and Gas Chromatography-Mass Spectrometry Analysis

Plant samples were pulverized to 5 μm using a grinding instrument (MM 400, Retsch GmbH, Haan, Germany), and 90 mg aliquots of roots, stems, leaves, and petioles were extracted. A mixed solution of 40 μL 2-chloro-L-phenylalanine (0.3 mg/mL, internal standard) in 360 μL methanol (pre-cooled at −20 °C) was prepared for metabolite extraction. Metabolites were extracted with the mixed solution by sonication for 30 min, after which they were sonicated again with a chloroform and water solution. Subsequently, the solution was centrifuged at 14,000 rpm (revolutions per minute). The supernatant was collected in a derivatized glass bottle, evaporated to dryness. A derivatization method oximation reaction was then performed to increase the volatility of the metabolites [30]. The dried residue was redissolved in 80 μL methoxyamine pyridine solution (15 mg/mL) and incubated at 37 °C (90 min). Subsequently, an 80 μL aliquot of *N*,*O*-bis(trimethylsilyl)trifluoroacetamide (BSTFA) (including 1% trimethyl-cholorosilane), and 20 μL were added to the mixture, vortexing for 2 min, which were then derivatized for 60 min in 70 °C. After pretreatment, the solution was centrifuged (12,000 rpm at 4 °C for 10 min), after which the supernatant was transferred to a glass vial for GC-MS analysis.

The GC-MS analysis was performed using a 7890A-5975C system (Agilent, Santa Clara, CA, USA). An Agilent HP-5 (5%-phenyl) -methyl polysiloxane non-polar column was used for GC-MS. The GC process was as follows: an initial temperature of 60 °C; oven temperature increments of 8 °C·min^−1^ to 125 °C, 4 °C·min^−1^ increments to 210 °C, 5 °C·min^−1^ to 270 °C, and 10 °C·min^−1^ to 305 °C; and a final holding temperature of 305 °C for 3 min. Mass spectra acquisition parameters were as follows: ion source temperature, 260 °C; filament bias, −70 V; mass range, *m*/*z* of 50–600; and acquisition rate, 20 spectra·sec^−1^ [30].

### 2.3. Phenolic Metabolite Extraction and Liquid Chromatography-Mass Spectrometry Targeted Analysis

Plant samples were pulverized to 5 μm using a grinding instrument (MM 400, Retsch GmbH), and 500 mg aliquots of roots, stems, leaves, and petioles were extracted. Samples were extracted with 80% ethanol in water (10 mL) containing 0.1 mg/L lidocaine (internal standard) by sonication twice, each for 45 min. The sample extractions were filtered, and the filtrates were merged. The filtrates were then dried under low pressure using a vacuum cavitation instrument. The resultant extracted material was dissolved in the mobile phase (1 mL) and filtered through 0.22 μm-diameter micropores. The purified solution was analyzed by ultra-performance liquid chromatography quadrupole time-of-flight mass spectrometry (UPLC/Q-TOF-MS).

Separation was performed on an Acquity UPLC BEH C18 column (1.7 µm, 2.1 mm × 50 mm) with a VanGuard precolumn (BEH C18, 1.7 µm, 2.1 × 5 mm; Waters, Shang-Hai, China) and maintained at 30 °C. The volume injected was 2 μL. Gradient elution was performed at a flow rate of 0.25 mL·min^−1^ using the following solvent system: 0.05% acetic acid-water (A), 0.05% acetic acid-acetonitrile (B); 5% B-95% B from 0–23 min; 95% B-5% B from 23–25 min; and 5% B from 25–31 min. Analyses were performed using a UPLC/Q-TOF-MS system (Waters). The MS conditions were set as follows: positive ion mode, capillary voltage of 3.0 kV, cone voltage of 45 V, source temperature of 400 °C, desolvation temperature of 500 °C, cone gas flow of 50 L/h, and desolvation gas flow of 800 L/h. Detection was performed in positive ion mode in the *m*/*z* range of 50–1000.

### 2.4. Multivariate Analysis

The GC-MS raw data were transformed into NetCDF format by data analysis software (Agilent GC-MS 5975) and were later processed using the software R. Each compound was displayed as the peak area normalized to that of the internal standard. For further analysis, the treated R output data were exported to Microsoft Excel. National Institute of Standards and Technology (NIST) 14 library was searched to compare the structures of the compounds with that of the NIST database. Compounds were then identified based on the retention index and mass spectra with already known compounds in the NIST library. Peak detection, retention time alignment, and library matching were performed using the TargetSearch package from Bioconductor (Solvusoft Corporation, Los Angeles, CA, USA) [64], after which the normalized data were imported into SIMCA-P version 11.0 software (Umetrics, Umea, Sweden) for multivariate statistical analysis. The supervised method of partial least-squares discriminant analysis (PLS-DA) was used to compare tissue-specific differences between control and UV-B treatment regarding the identification of significant metabolites, and *t*-test combinatory approaches were used to screen for important metabolites (*p* < 0.05). The LC-MS data were analyzed using the software MassLynx version 4.1. This software detected peaks and listed the detected and matched peaks with the retention time and *m*/*z* pair and their corresponding intensities. The relative signal intensities of compounds were standardized by dividing them by the intensities of internal standard. The relationships between 15 primary metabolites and phenolic compounds were used for hierarchical clustering analysis (HCA) by R (www.r-project.org/) for both species. Pearson’s correlation coefficients were calculated for these metabolites, and the Tukey test was performed using Statistical Product and Service Solutions (SPSS) version 17.0. Metabolic pathways were analyzed using the Metaboanalyst web portal (www.metaboanalyst.ca) and MBRole (http://csbg.cnb.csic.es/mbrole). The pathways of metabolites were analyzed using database sources including the Kyoto Encyclopedia of Genomes and Genomes (KEGG) (http://www.genome.jp/kegg/) to identify the most affected metabolic pathways and facilitate further metabolite interpretation. The metabolites and corresponding pathways were imported into Cytoscape software version 3.1.0 to visualize the network models. A metabolic correlation distribution network was created from the 144 primary metabolite data using the WGCNA package (Solvusoft Corporation, Los Angeles, CA, USA) [65,66].

### 2.5. RNA Extraction and Real-Time Polymerase Chain Reaction Analysis

The extraction and derivatization of RNA from roots, leaves, stems and petioles were performed as described previously by Liu et al. [67]. Amplification, detection, and data analysis were performed using a Rotor-Gene 6000 real-time rotary analyzer (Corbett Life Science, Sydney, Australia). Primer sequences for PCR were as follows (Table 1):

To determine relative fold differences in template abundance for each sample, the cycle threshold (Ct) values for each of the gene-specific primers were normalized to the Ct value for *18S* RNA.

## 3. Results

### 3.1. Morphological and Physiological Changes Induced by Ultraviolet-B Radiation

**Figure 1:**
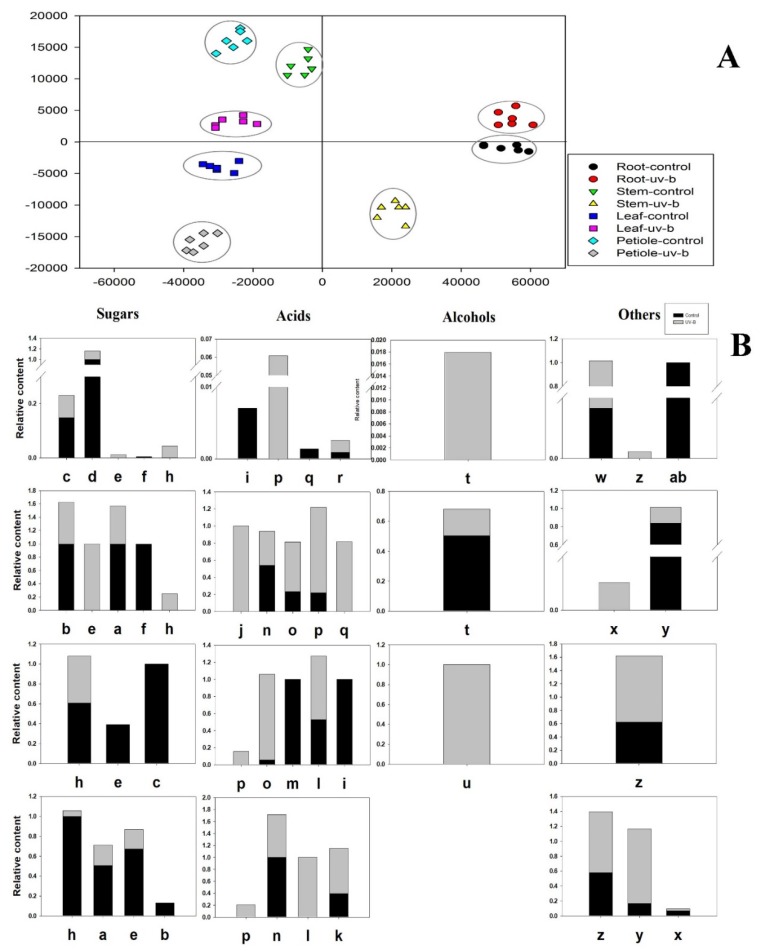
Metabolic analysis of specific tissues of *A. mongholicus* under control and UV-B treatments analyzed by GC-MS. (**A**) Partial least-squares discriminant analysis (PLS-DA); (**B**) The expression pattern of potential differences. **a**, mannose; **b**, 3-mannobiose; **c**, fructose; **d**, talose; **e**, glucose; **f**, psicose; **g**, sorbofuranose; **h**, myo-inositol; **i**, 2,3,4-trihydroxybutyric acid; **j**, 4-aminobutyric acid; **k**, citric acid; **l**, hexonic acid; **m**, isothiocyanic acid; **n**, malic acid; **o**, oxalic acid; **p**, phosphonic acid; **q**, palmitic acid; **r,** propanedioic acid; **s**, stearic acid; **t**, pinitol; **u**, erythritol; **w**, d-glucopyranoside; **x**, 2-*O*-glycerol-d-galactopyranoside; **y**, methyl galactoside; **z**, glycerol; **ab**, dotriacontane; **ac**, 1-monopalmitin.

The effect of UV-B radiation on morphology can be seen in Figure 1 and Table 2. Compared with the control group, stem growth was relatively short and the leaf more compact under UV-B radiation. Both plant height and leaf area decreased under UV irradiation. The whole-plant proportion of roots (*n* = 10) was 19.07% for the control group and 20.70% for the UV-B treatment group. In contrast to the control group, the chlorophyll content decreased in the UV-B treated group. The activity of antioxidant enzymes and content of H_2_O_2_ increased under UV-B radiation.

### 3.2. Primary Metabolism Reprogramming between the below- and Aboveground Organs in Response to Ultraviolet-B Radiation

In the present study, approximately 184 metabolites comprising sugars, acids, alcohols, and other compounds were identified using a GC-MS platform in four tissues of *A. mongholicus* under control and UV-B treatments (Supplementary Table S1).

To identify the metabolites that contributed to these variances, 27 significantly differential compounds were identified by their variables of importance in the projection (VIP) values >1, and their *p*-values < 0.05 (Figure 1B). There were eight significant differences regarding sugars; most of the sugars were monosaccharides. The 3-α-mannobiose was the only disaccharide that significantly changed under UV-B, and its monosaccharide (mannose) constituents have a common tendency to decrease in aboveground tissue (stems and petioles). In contrast to the control, the levels of d-glucose and myo-inositol increased in roots and stems but decreased in leaves and petioles under UV-B treatment. The d-talose was significantly reduced by UV-B radiation in belowground tissues (roots) but did not significantly change in aboveground tissues. Regarding acids, including eight organic acids, one inorganic acid and two fatty acids, diverse trends were observed. Phosphonic acid level decreased under UV-B radiation in all organs. Propanedioic acid increased only in roots, and there was no significant difference in aboveground tissue under control or UV-B treatments. Palmitic acid decreased in belowground tissue (roots) but increased in aboveground tissues (stems) under UV-B radiation. Oxalic acid increased in leaves and stems while hexonic acid increased in leaves and petioles under UV-B radiation. Significant increases in alcoholic compounds in belowground tissues (roots) were observed for d-pinitol, whereas significant increases in aboveground tissues (leaves) were observed for erythritol. In addition, the levels of six other compounds significantly differed from those of the control group under UV-B radiation. Among these compounds, α-d-glucopyranoside levels significantly differed only in belowground tissues (roots).

The PLS-DA was used to profile these metabolites. In the PLS-DA score plot, separation of the two treatments and their different tissues was obtained. The samples were clustered into separate groups (Figure 1A). A PLS-DA model was created with two principal components: PLS1 (47.9%) and PLS2 (64.7%). A clear classification trend was observed among roots, stems, leaves, and petioles for all samples in the score plot. The control and UV-B treatment groups of the aboveground organs (stems, leaves and petioles) were better separated than those of the belowground organs (roots).

### 3.3. Phenolic Compounds Were Concentrated in Leaves Compared to Roots under Ultraviolet-B Radiation

**Figure 2:**
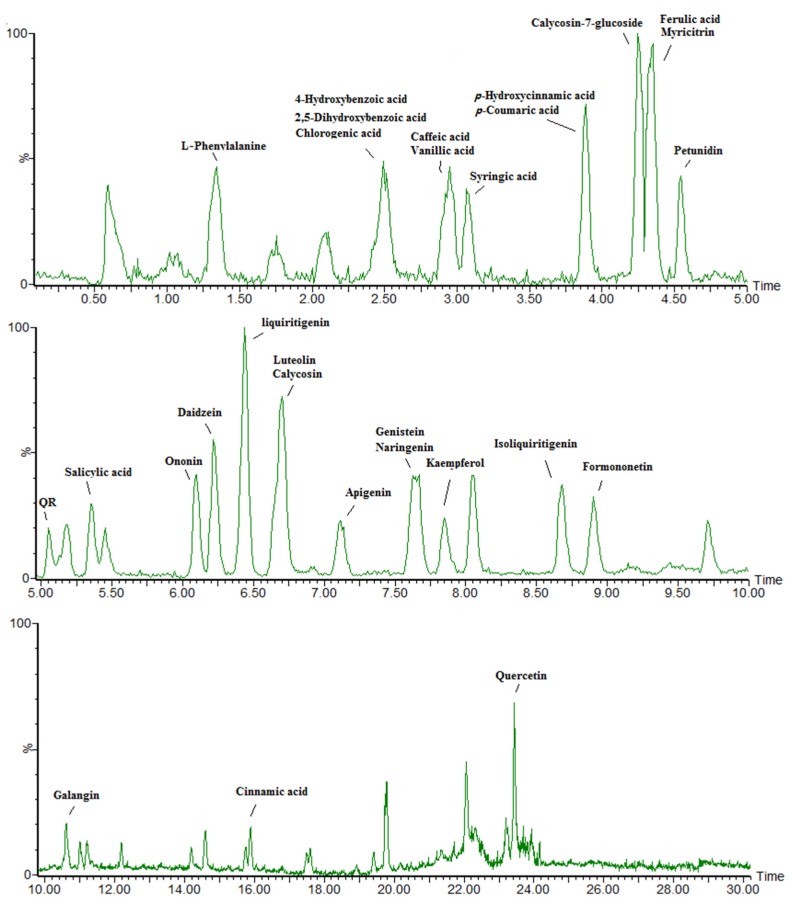
Chromatogram of the target compounds from ultra-performance liquid chromatography quadrupole time-of-flight mass spectrometry (UPLC-TOF-MS). Targeted compounds in samples were determined in accordance with the mass spectral information and retention time of the corresponding standard reference compounds. QR: Quercetin-3-*O*-rhamnoside.

To understand the effects of increased UV-B radiation on the level of phenolic metabolites, the targeted phenolic compounds content of the collected samples was analyzed via LC-MS. A total of 29 standard reference compounds were used, all provided by Shanghai yuanye Bio-Technology Co. Ltd (ShangHai, China), with a purity of ≥98%. The targeted compounds in the samples were identified in accordance with the mass spectral information and retention time of the corresponding standard reference compounds. The mass spectral information has been added as Supplementary Table S2; the retention time and corresponding standard reference compounds were shown in the chromatogram of Figure 2. In total, 29 phenolic metabolites were analyzed.

**Figure 3:**
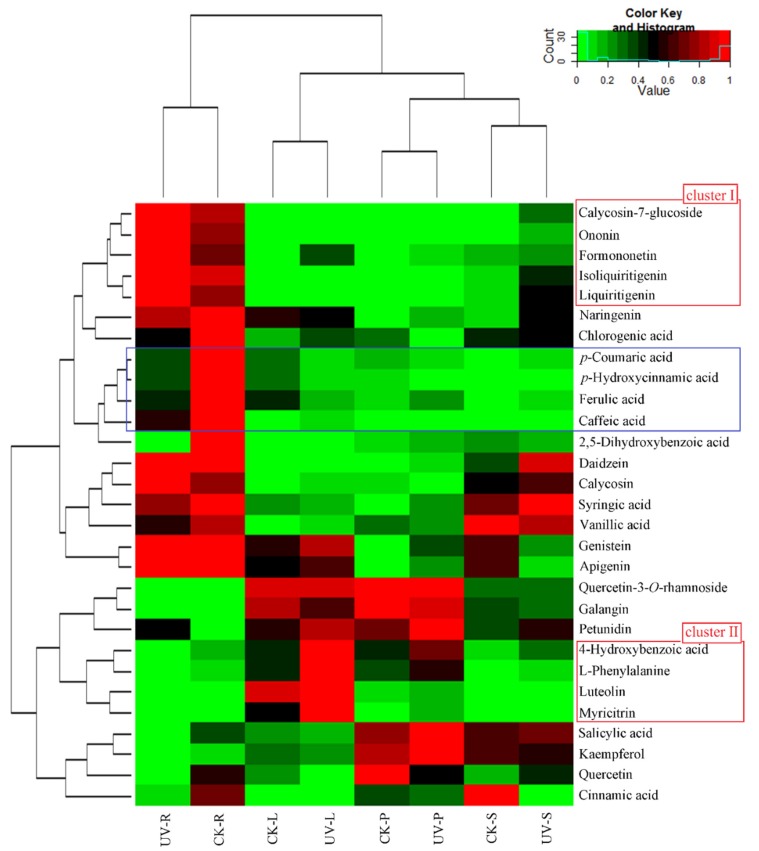
Distribution of potential biomarkers in specific tissues from the control and UV-B treatment groups. Heat map visualization of the relative differences of potential biomarkers in the different samples. The data of the content value of each compound were normalized to complete the linkage hierarchical clustering. Each tissue type is visualized in a single column, and each metabolite is represented by a single row: CK-R, CK-S, CK-L, and CK-P correspond to roots, stems, leaves, and petioles of the control group; UV-R, UV-S, UV-L, and UV-P correspond to roots, stems, leaves, and petioles from the UV-B-treated group. Red indicates high abundance of compounds, whereas green indicates low abundance (color key scale above heat map).

To further clarify phenolic accumulation patterns in different tissues under the control and UV-B treatments, the roots, stems, leaves, and petioles of *A. mongholicus* were determined separately. Visualization of the phenolic profile was performed using HCA (Figure 3). The accumulation of phenolics displayed a clear phenotypic variation in terms of phenolic abundance in the different tissues and treatments. Roots contained the highest levels of the majority of tested phenolics, followed by the leaves, petioles and stems. Based on their tissue- and UV-B specific accumulation patterns, phenolics clearly grouped into two clusters.

**Figure 4:**
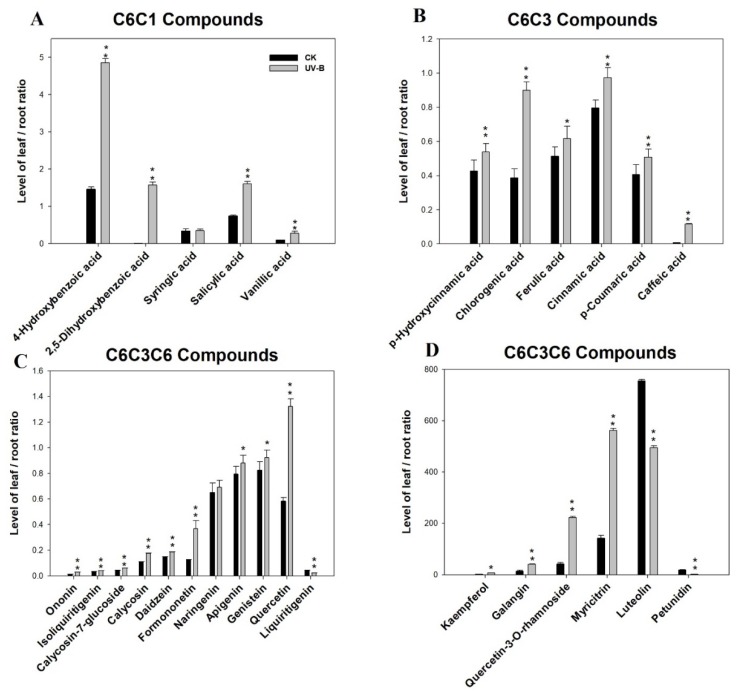
Distribution of phenolics that have various carbon skeletons in above- and belowground tissues under UV-B radiation. The ordinate is set to the ratio of compound contents in leaves and roots. (**A**) Phenolics with C6C1 carbon skeletons; (**B**) phenolics with C6C3 carbon skeletons; (**C**) and (**D**) phenolics with C6C3C6 carbon skeletons.

Phenolics in cluster I showed higher levels in the roots of the UV-B treatment group than in those of the control group and mostly consisted of calycosin-7-glucoside, ononin, formononetin, isoliquiritigenin, and liquiritigenin. Phenolics in cluster II consisted of 4-hydroxybenzoic acid, l-phenylalanine, luteolin and myricitrin; higher levels of these compounds were detected in the leaves of the UV-B radiation group than in those of the control group. In addition, UV-B radiation altered the distribution of phenolic compounds between above- and belowground tissues (Figure 4). The ordinate of Figure 4 is set to the ratio of compound contents in leaves and roots.

According to Figure 4, the majority of the ratios of phenolic levels in leaves/roots under UV-B treatment were higher than those of the control. In other words, compared with those of the control, phenolic compounds were more concentrated in leaves under UV-B radiation. Moreover, all the C6C3 carbon skeleton phenolics were translocated from the roots to the leaves.

### 3.4. Construction of an Integrative View of the Primary and Phenolic Metabolite Network for Specific Tissues and Growth Conditions

**Figure 5:**
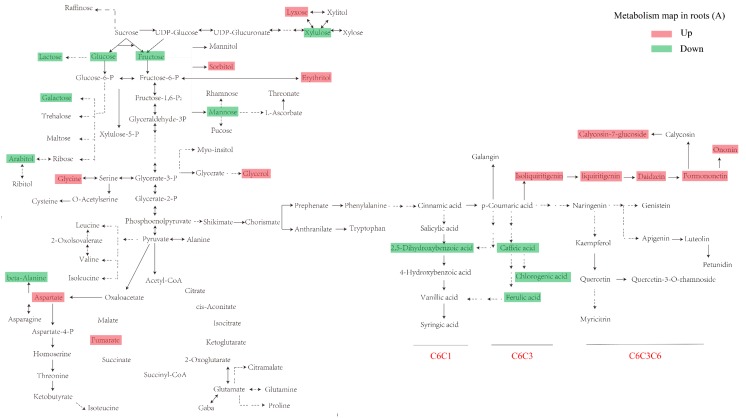

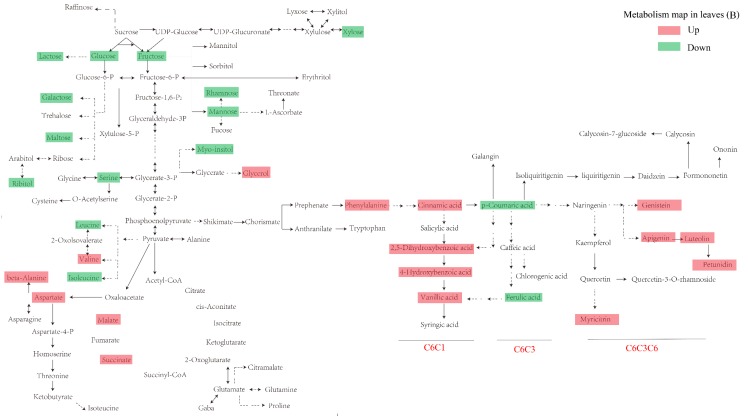
Visualization of primary and phenolic metabolites dynamics on a biochemical pathway map. (**A**) Metabolism map in roots. (**B**) Metabolism map in leaves. Compounds in the red box are upregulated by UV-B radiation, and compounds in the green box are downregulated.

To provide better overview of these data, a simplified primary and phenolic metabolites pathway was used to show the metabolic responses to UV-B radiation among different tissues. As shown in Figure 5A,B, for primary metabolites, the accumulation of soluble sugars, such as glucose, fructose, and mannose, decreased in response to UV-B radiation in both roots and leaves. In difference with leaves the level of erythrytiol and sorbitol increased in the roots. Some amino acids including valine and aspartate increased in response to UV-B radiation in both leaves and roots. The levels of acids involved in the tricarboxylic acid (TCA) cycle, including fumarate in roots, malate, and succinate in leaves, increased in the UV-B treatment group. For phenolic metabolites, most of the isoflavones in roots were upregulated by UV-B induction. Unlike roots, the upregulated phenolic metabolites in leaves mainly belong to phenolic acids and flavones. We only observed that the C6C3 phenolics decreased both in roots and leaves. In addition, phenylalanine, which is the key node in the synthesis of phenolic compounds from the shikimate pathway, may be responsible for the increase in the majority of phenolic compounds under UV-B radiation.

### 3.5. Expression of Genes Involved in Isoflavonoids Pathway in Leaves and Roots

**Figure 6:**
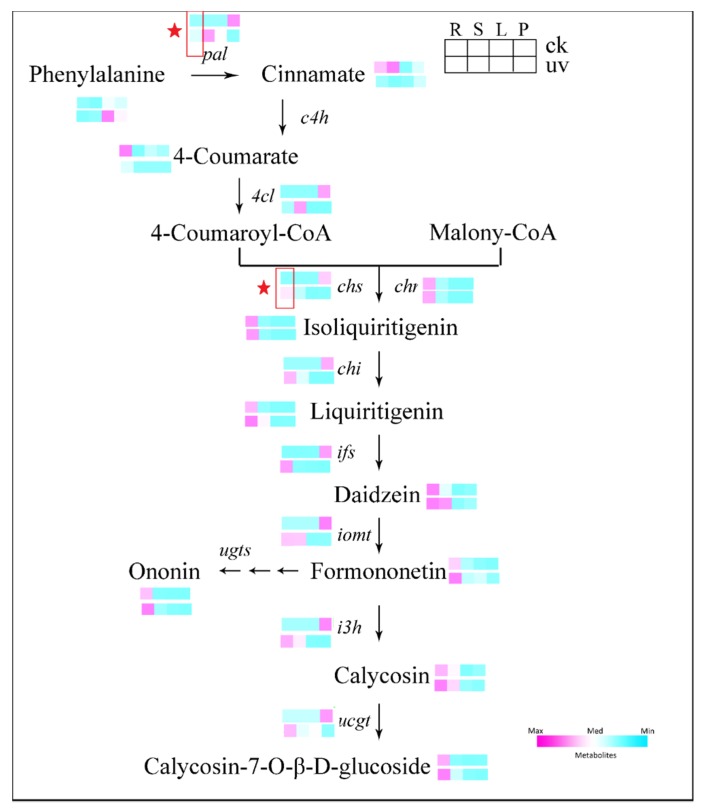
Visualization of isoflavonoid metabolites and relative gene dynamics on a biochemical pathway map. The levels of isoflavonoid metabolites and relative genes were both averaged over three biological replicates after normalization. Within each box, each column is a specific tissue (from left to right: root, R; stem, S; leaf, L; petiole, P), as shown in the upper-right corner. The intensity of the ratio of the UV-B group to the control (CK) group is indicated by the color scale key in the upper-right corner.

To further explore the metabolic mechanisms of CAG in the enhanced UV-B environment, the CAG biosynthesis pathway and the expression levels of relevant genes were visualized, as shown in Figure 6. The expression of the genes encoding enzymes that directly participate in the formation of CAG in samples collected from different treatments and different tissues was analyzed using RT-PCR. Gene expression levels were normalized using the *18S* RNA reference gene as an internal standard. The transcription level of the synthetase genes involved in the CAG pathway, including *CHI*, *IFS*, *IOMT*, *I3′H*, and *UCGT*, were upregulated in roots but downregulated in aboveground tissues in response to UV-B induction. This variation is strikingly similar to the accumulation pattern of their corresponding compounds. The *CHS* and *CHR* co-catalyze the condensation of *p*-coumaryl-CoA with three malonyl-CoA molecules toward the formation of the isoliquiritigenin, an isoflavonoid skeleton. The increased expression of *CHS* was induced by UV-B in the roots. Compared with *CHR*, *CHS* is clearly more responsive to UV-B.

## 4. Discussion

Increased UV-B radiation affects plant development, morphology, physiology and metabolism. At the same time, environmental vicissitude will greatly impact *A. mongholicus* in the field. Therefore, the metabolic response of *A. mongholicus* to UV-B strongly influences the quality of Radix astragali that is provided to the market. In this study, we used untargeted GC-MS and targeted LC-MS techniques at primary and secondary compound levels to investigate the abundance and identity of compounds, revealing different metabolic profiles for specific tissues under different conditions. To explore the reprogramming of primary and secondary metabolic responses to UV-B and the formation of adaptive stable metabolism in above- and under- ground tissues. We also evaluated the level of isoflavones and their related genes to explore the mechanism of CAG accumulation under UV-B.

Based on the observation of morphological indicators and the detection of physiological indicators (Figure 1 and Table 1), leaves were more compact and plant height, leaf area, and chlorophyll content were reduced. These results indicate that growth was inhibited and that energy might be transferred to the accumulation of more functional metabolites to adapt to the changing environment [68]. The relative root biomass increased slightly, suggesting that energy was transferred to the roots in response to the UV-B radiation. Moreover, an increase antioxidant enzymes and H_2_O_2_ (Table 1) indicated that the stress response of plants to UV-B was actively operating [69,70].

We based our study on GC-MS-metabolomics to analyze tissue-specific changes under UV-B radiation and control conditions of *A. mongholicus*. Sugars, which are energy sources for growth, decreased in aboveground tissues but increased in belowground tissues. This explains the decrease in plant height and leaf area and the increase in root specific gravity. Additionally, the reduction in glucose in the leaves could indicate that a stronger shift from carbon assimilation to carbon accumulation occurred after UV-B radiation. Moreover, increased levels of sugar alcohols (e.g., erythritol) derived from the pentose phosphate pathway (PPP) were also observed in the UV-B treatment group, likely reflecting the higher respiratory rate of the PPP pathway in these plants. In addition, PLS-DA plots of the primary metabolites for four tissues under different conditions were generated (Figure 1A). The analysis of the score plots revealed a clear clustering of biological replicates for each sample.

The dynamic patterns of targeted phenolic compounds in four tissues of control and UV-B treatment plants was detected by UPLC-q/TOF-MS. Interestingly, the content of flavanones and flavonols increased in the leaves in response to UV-B radiation, whereas the isoflavonoids increased in the roots. Several early physiological experiments provided circumstantial evidence that phenolics are involved in UV-B protection [71]. Among these phenolics, flavones and flavonols protect cells because these compounds accumulate in the epidermal layers of leaves and stems, acting as filters and absorbing radiation in the UV-B portion of the spectrum [72,73]. Ryan et al. reported that UV radiation induces the synthesis of flavonols that have higher hydroxylation levels in *Petunia* and *Arabidopsis*. Those authors suggested that flavonols may play as yet uncharacterized roles in the UV stress response because flavonols have UV-absorbing properties, and the hydroxylation of flavonols might positively affect antioxidant capacity [74,75]. Regarding compounds involved in UV-B protection, the structures of flavones and flavonols are the most advantageous [15]. Thus, the most significant changes occurred regarding the levels of flavone and flavonol compounds (such as luteolin and myricitrin) under UV-B radiation were in leaves. At the same time, the accumulation of isoflavonoids (such as calycosin-7-glucoside, ononin, and formononetin) in roots constitutes a reserve of active phenolics that can easily be mobilized at any given time, especially under UV-B stress conditions. Furthermore, a common feature of the changes in phenolic compounds under UV-B exposure in roots and leaves was observed. The C6C3C6 carbon compounds increasingly accumulated at the expense of C6C3 carbon compounds such as chlorogenic acids, ferulic acid, and cinnamic acid. In view of this phenomenon, the increase in C6C3C6 carbon compounds (flavone and flavonol in the leaves and isoflavonoid in the roots) at the cost of C6C3 carbon compounds may be due to the stronger UV-B absorptive and antioxidant capacity of C6C3C6 carbon compounds than that of C6C3 ones in *A. mongholicus*. Under UV-B radiation, the phenolic compounds of various carbon skeletons are concentrated in the leaves (Figure 4), probably because the leaves are most directly affected by UV-B radiation. The decreasing of sugars and enrichment of phenolics in leaves could also result in reduced energy for growth and reduced leaf area.

To obtain a more detailed overview regarding the tissue-specific differences of the identified metabolites between the UV-B and control treatments, we built a primary and phenolic metabolites network (Figure 5A,B). The TCA cycle intermediates such as succinate and fumarate, which are major regulators of carbon and nitrogen interactions, increased under UV-B radiation. At the same time, this increase also occurred for shikimate and L-phenylalanine. It seems highly likely that shikimate would be elevated following the mobilization of carbon reserves stored in plants to increase the flux through the phenylpropanoid pathway under UV-B radiation [76]. In plants, phenylalanine is thought to be the general precursor of C6C1-, C6C3-, and C6C3C6 compounds and their polymers such as tannins and lignins [77]. As such, when considered together, these data suggest that in response to UV-B the plant cell is “primed” at the level of primary metabolism by a mechanism that involves the reprogramming of metabolism to efficiently divert carbon toward the aromatic amino acid precursors of the phenylpropanoid pathway [78]. Tryptophan and phenylalanine compete for chorismate to synthesize alkaloids and phenols, respectively. The results showed more accumulation of phenylalanine under UV-B radiation. This suggests that the increase in phenolic compounds under UV-B radiation is caused by the transfer of carbon from primary metabolism and involves metabolic reprogramming. For phenylpropanoid pathway under UV-B radiation, a significant difference appears between the above- and the underground tissues. Flavones were significantly increased in leaves, probably because of the roles of flavones accumulation in the epidermal layers of leaves acting as filters and absorbing radiation in the UV-B portion of the spectrum [79]. The phenolic acids enhanced accumulation in leaves might be acting as antioxidant supplements. Different from the metabolic response in leaves, enhanced accumulation of isoflavones were observed in roots. These isoflavones might constitute a reserve of active phenolics that can easily be mobilized at any given time, especially under UV-B stress conditions. Phenylalanine, as a key node in phenolic metabolism, showed a significant increase only in leaves under UV-B radiation. A bold and reasonable assumption is that the upregulation of the relevant genes rather than the synthetic precursor (phenylalanine) might be the most important contributor for the activation of phenolic metabolism in the roots.

In order to verify the possibility of this assumption for further, and due to the therapeutic potential of these isoflavones (especially CAG), the metabolic mechanisms of these compounds in the enhanced UV-B environment need to be further explored. The CAG biosynthesis pathway and relevant genes are visualized in Figure 6. The promoters of a series of genes involved in the flavonoid biosynthetic pathway contain a specific recognized domain that can interact with the MYB family of transcription factors via light-responsive elements [80]. In the present study, targeted isoflavonoid biosynthetic genes have transcriptional activation; as suggested, these transcription factors could be activated by UV-B radiation and could affect the investigated genes (Figure 6). In general, *PAL* genes have been proposed as a dominant control point of phenylpropanoids, flavonoids, and isoflavonoids biosynthesis in response to various biotic and abiotic stresses inclusive of pathogen attack, UV radiation, and mechanical wounding [81,82]. The no-significant of phenylalanine and increased transcription levels of *PAL* indicates that the response of phenolics in roots to UV-B is activated by relevant genes rather than phenylalanine. The increased transcription levels of *PAL* in roots, stems, and leaves indicated that the response of phenolics to UV-B is stimulated in different tissues. *CHS* and *CHR* co-catalyze the condensation of *p*-coumaryl-CoA with three malonyl-CoA molecules toward the formation of the Isoliquiritigenin, an isoflavonoid skeleton. The increased expression of *CHS* was induced by UV-B in the roots. Compared with *CHR*, *CHS* is clearly more responsive to UV-B, which suggests that the increased accumulation of isoflavonoids might be due to the elevated levels of *CHS* in the UV-B environment. The transcription level of the synthetase genes involved the CAG pathway, including *CHI*, *IFS*, *IOMT*, *I3′H*, and *UCGT*, were upregulated in the roots in response to UV-B induction but downregulated in aboveground tissues. This variation is strikingly similar to the accumulation pattern of the corresponding compounds of these enzymes. These results suggest that in the enhanced UV-B environment, tissue-specific increases in the levels of active isoflavones such as CAG are due to the regulation of the elevated levels of synthesis genes in the roots.

## 5. Conclusions

A metabolic profile was revealed using untargeted GC-MS and targeted LC-MS combined techniques to investigate the tissue-specific metabolic mechanism of *A. mongholicus* in an enhanced UV-B environment. We found that in response to UV-B the plant cell is “primed” at the level of primary metabolism by a mechanism that involves the reprogramming of metabolism to efficiently divert carbon toward the aromatic amino acid precursors of the phenylpropanoid pathway. A stronger shift from carbon assimilation to carbon accumulation has occurred. Among the accumulation of phenolics, C6C1 carbon compounds (phenolic acids in leaves) and C6C3C6 carbon compounds (flavones and flavonols in leaves and isoflavones in roots) increased at the expense of C6C3 carbon compounds in order to obtain the stronger UV-B absorptive and antioxidant capacity. Compared with the control treatment, the response of phenolics has a significant tissue-specific in the UV-B radiation treatment. Notably, the response of phenolics in roots to UV-B is activated by upregulation of relevant genes rather than phenylalanine.

## Figures and Tables

**Table 1:** Primer sequences for polymerase chain reaction (PCR).

Gene Name		Primer Sequence (5′ to 3′)
*CHS*	Forward	CCTTCTTTGGATGCTAGACAAGACA
	Reverse	CGAAGACCCAAGAGTTTGGTTAGTT
*PAL*	Forward	CATCAAATCTCTCTGGCAGTAGGAA
	Reverse	AGTTCACATCTTGGTTATGCTGCTC
*C4H*	Forward	AACAAAGTGAGGGATGAAATTGACA
	Reverse	GGATTGCCATTCTTAGCCTTAGTGT
*4CL*	Forward	TGTCCCTCCTATTGTTTTGGCTATT
	Reverse	CTTTGGGGAATTTAGCTCTGACAGT
*CHR*	Forward	AAACAAGGTTACAGGCATTTTGACA
	Reverse	GGAAGAACGAGATGAGGATGATTTT
*CHI*	Forward	ATCGAGTTTTTCCACCAGGATCTAC
	Reverse	ATCATAGTCTCCAACACAGCCTCAG
*IFS*	Forward	CCTTCACCTATTGGACAAACCTCTT
	Reverse	CCTGGTATTAAAGGAAGAAGCCTCA
*IOMT*	Forward	GCACAAAACACAAGATCAAACTTC
	Reverse	GCATTACGGCCATTGATTG
*I3′H*	Forward	GGATGTTAAAGAAGCGAAGCAATTT
	Reverse	ATCAAACAATCTCAACAAAGGCAAA
*UCGT*	Forward	AGGTTTTGAAGATTTATGCACCA
	Reverse	TCCTTTCTGAGTTCCAGGACA
*18S*	Forward	TGCAGAATCCCGTGAACCATC
	Reverse	AGGCATCGGGCAACGATATG

**Table 2:** Morphological and physiological indices of control and Ultraviolet treatments (*n* = 20).

	Height (cm)	Root/Whole Fresh Weight (FW) (%)	Leaf Area (cm^2^)	Chlorophyll (mg·g^−1^)
Control	24.2 ± 0.6	19.07 ± 0.31	0.292 ± 0.004	1.242 ± 0.008
UV-treated	23.4 ± 0.6	20.7 ± 0.67	0.230 ± 0.006	0.799 ± 0.011
	**CAT** **(U·g^−1^·min^−1^, FW)**	**POD** **(U·g^−1^·min^−1^, FW)**	**APX** **(U·g^−1^·min^−1^, FW)**	**H_2_O_2_** **(μmoL·g^−1^)**
Control	26.25 ± 0.01	1800 ± 1	0.06 ± 0.001	3.59 ± 0.01
UV-treated	36.75 ± 0.01	7050 ± 1	0.15 ± 0.003	3.79 ± 0.01

Abbreviations: CAT: catalase; POD: peroxidase; APX: ascorbate peroxidase; H_2_O_2_: hydrogen peroxide.

## Supplementary Materials

The following are available online at https://www.mdpi.com/2218-273X/10/4/504/s1, Table S1: Compounds identified in *Astragalus mongholicus* measured by Gas Chromatography-Mass Spectrometry; Table S2: Metabolite reporting checklist and recommendations for Liquid Chromatography-Mass Spectrometry.

## References

[1] Pan H., Fang C., Zhou T., Wang Q., Chen J. (2007). Accumulation of calycosin and its 7-*O*-beta-D-glucoside and related gene expression in seedlings of Astragalus membranaceus Bge. var. mongholicus (Bge.) Hsiao induced by low temperature stress. Plant Cell Rep..

[2] Napolitano A., Akay S., Mari A., Bedir E., Pizza C., Piacente S. (2013). An analytical approach based on ESI-MS, LC–MS and PCA for the quali–quantitative analysis of cycloartane derivatives in Astragalus spp. J. Pharm. Biomed. Anal..

[3] Kuo Y.-H., Tsai W.-J., Loke S.-H., Wu T.-S., Chiou W.-F. (2009). Astragalus membranaceus flavonoids (AMF) ameliorate chronic fatigue syndrome induced by food intake restriction plus forced swimming. J. Ethnopharmacol..

[4] Wu J.-H., Li Q., Wu M.-Y., Guo D.-J., Chen H.-L., Chen S.-L., Seto S.W., Au A.L., Poon C.C., Leung G.P.-H. (2010). Formononetin, an isoflavone, relaxes rat isolated aorta through endothelium-dependent and endothelium-independent pathways. J. Nutr. Biochem..

[5] Fan Y., Wu D.-Z., Gong Y.-Q., Zhou J.-Y., Hu Z.-B. (2003). Effects of calycosin on the impairment of barrier function induced by hypoxia in human umbilical vein endothelial cells. Eur. J. Pharmacol..

[6] Cho W.C.S., Leung K.N. (2007). In vitro and in vivo immunomodulating and immunorestorative effects of Astragalus membranaceus. J. Ethnopharmacol..

[7] Cho W.C., Leung K.N. (2007). In vitro and in vivo anti-tumor effects of Astragalus membranaceus. Cancer Lett..

[8] Ma X., Tu P., Chen Y., Zhang T., Wei Y., Ito Y. (2003). Preparative isolation and purification of two isoflavones from Astragalus membranaceus Bge. var. mongholicus (Bge.) Hsiao by high-speed counter-current chromatography. J. Chromatogr. A.

[9] Nakamura T., Hashimoto A., Nishi H., Kokusenya Y. (1999). Investigation on the Marker Substances of Crude Drugs in Formulations. I.: Marker Substances for the Identification of Astragali Radix in Kampo and Drinkable Preparations. Yakugaku Zasshi.

[10] Yu O., Shi J., Hession A.O., Maxwell C.A., Mcgonigle B., Odell J.T. (2003). Metabolic engineering to increase isoflavone biosynthesis in soybean seed. Phytochem..

[11] Frohnmeyer H., Staiger D. (2003). Ultraviolet-B Radiation-Mediated Responses in Plants. Balancing Damage and Protection. Plant Physiol..

[12] Jansen M.A.K., Gaba V., Greenberg B.M. (1998). Higher plants and UV-B radiation: Balancing damage, repair and acclimation. Trends Plant Sci..

[13] Rozema J., Van De Staaij J., Björn L.O., Caldwell M. (1997). UV-B as an environmental factor in plant life: Stress and regulation. Trends Ecol. Evol..

[14] Kusano M., Tohge T., Fukushima A., Kobayashi M., Hayashi N., Otsuki H., Kondou Y., Goto H., Kawashima M., Matsuda F. (2011). Metabolomics reveals comprehensive reprogramming involving two independent metabolic responses of Arabidopsis to UV-B light. Plant J..

[15] Matsuura H.N., De Costa F., Yendo A.C.A., Fett-Neto A.G. (2012). Photoelicitation of Bioactive Secondary Metabolites by Ultraviolet Radiation: Mechanisms, Strategies, and Applications. Biotechnol. Med. Plants.

[16] Hahlbrock K., Scheel D. (1989). Physiology and Molecular Biology of Phenylpropanoid Metabolism. Annu. Rev. Plant Biol..

[17] Frolov A., Henning A., Böttcher C., Tissier A., Strack D. (2013). An UPLC-MS/MS Method for the Simultaneous Identification and Quantitation of Cell Wall Phenolics in Brassica napus Seeds. J. Agric. Food Chem..

[18] Parr A.J., Bolwell G.P. (2000). Phenols in the plant and in man. The potential for possible nutritional enhancement of the diet by modifying the phenols content or profile. J. Sci. Food Agric..

[19] Beckman C.H. (2000). Phenolic-storing cells: Keys to programmed cell death and periderm formation in wilt disease resistance and in general defence responses in plants?. Physiol. Mol. Plant Pathol..

[20] Valcarcel J., Reilly K., Gaffney M., O’Brien N.M. (2015). Antioxidant Activity, Total Phenolic and Total Flavonoid Content in Sixty Varieties of Potato (Solanum tuberosum L.) Grown in Ireland. Potato Res..

[21] García B.A., Berrueta L.A., Garmón-Lobato S., Gallo B., Vicente F. (2009). A general analytical strategy for the characterization of phenolic compounds in fruit juices by high-performance liquid chromatography with diode array detection coupled to electrospray ionization and triple quadrupole mass spectrometry. J. Chromatogr. A.

[22] Caravaca G., López-Cobo A., Verardo V., Segura-Carretero A., Gutierrez A.F. (2016). HPLC-DAD-Q-TOF-MS as a powerful platform for the determination of phenolic and other polar compounds in the edible part of mango and its by-products (peel, seed and seed husk). Electrophoresis.

[23] Herrmann K.M., Weaver L.M. (1999). The shikimate pathway. Annu. Rev. Plant Boil..

[24] Maeda H., Dudareva N. (2012). The Shikimate Pathway and Aromatic Amino Acid Biosynthesis in Plants. Annu. Rev. Plant Boil..

[25] Babst B.A., Harding S.A., Tsai C.-J. (2010). Biosynthesis of Phenolic Glycosides from Phenylpropanoid and Benzenoid Precursors in Populus. J. Chem. Ecol..

[26] Wink M. (2010). Biochemistry of Plant Secondary Metabolism.

[27] Marković S., Tošović J. (2015). Application of Time-Dependent Density Functional and Natural Bond Orbital Theories to the UV–vis Absorption Spectra of Some Phenolic Compounds. J. Phys. Chem. A.

[28] Song J.-Z., Mo S.-F., Yip Y.-K., Qiao C.-F., Han Q.-B., Xu H. (2007). Development of microwave assisted extraction for the simultaneous determination of isoflavonoids and saponins in radix astragali by high performance liquid chromatography. J. Sep. Sci..

[29] Wu T., Bligh S.A., Gu L.-H., Wang Z.-T., Liu H.-P., Cheng X.-M., Branford-White C.J., Hu Z.-B. (2005). Simultaneous determination of six isoflavonoids in commercial Radix Astragali by HPLC-UV. Fitoterapia.

[30] Liu J., Liu Y., Wang Y., Abozeid A., Yuan-Gang Z., Tang Z.-H. (2017). The integration of GC–MS and LC–MS to assay the metabolomics profiling in Panax ginseng and Panax quinquefolius reveals a tissue- and species-specific connectivity of primary metabolites and ginsenosides accumulation. J. Pharm. Biomed. Anal..

[31] Park H.-W., In G., Kim J.-H., Cho B.-G., Han G.-H., Chang I.-M. (2013). Metabolomic approach for discrimination of processed ginseng genus (Panax ginseng and Panax quinquefolius) using UPLC-QTOF MS. J. Ginseng Res..

[32] Li L., Luo G.-A., Liang Q., Hu P., Wang Y.-M. (2010). Rapid qualitative and quantitative analyses of Asian ginseng in adulterated American ginseng preparations by UPLC/Q-TOF-MS. J. Pharm. Biomed. Anal..

[33] Fernie A.R., Trethewey R.N., Krotzky A.J., Willmitzer L. (2004). Metabolite profiling: From diagnostics to systems biology. Nat. Rev. Mol. Cell Boil..

[34] Fiehn O. (2001). Combining Genomics, Metabolome Analysis, and Biochemical Modelling to Understand Metabolic Networks. Comp. Funct. Genom..

[35] Obata T., Fernie A.R. (2012). The use of metabolomics to dissect plant responses to abiotic stresses. Cell. Mol. Life Sci..

[36] Ye G., Zhu B., Yao Z., Yin P., Lu X., Kong H., Fan F., Jiao B., Xu G. (2012). Analysis of Urinary Metabolic Signatures of Early Hepatocellular Carcinoma Recurrence after Surgical Removal Using Gas Chromatography–Mass Spectrometry. J. Proteome Res..

[37] Li Y., Ruan Q., Li Y., Ye G., Lü X., Lin X., Xu G. (2012). A novel approach to transforming a non-targeted metabolic profiling method to a pseudo-targeted method using the retention time locking gas chromatography/mass spectrometry-selected ions monitoring. J. Chromatogr. A.

[38] Lisec J., Schauer N., Kopka J., Willmitzer L., Fernie A.R. (2006). Gas chromatography mass spectrometry–based metabolite profiling in plants. Nat. Protoc..

[39] Lisec J., Schauer N., Kopka J., Willmitzer L., Fernie A.R. (2015). Corrigendum: Gas chromatography mass spectrometry-based metabolite profiling in plants. Nat Protoc..

[40] Schauer N., Steinhauser D., Strelkov S., Schomburg D., Allison G., Moritz T., Lundgren K., Roessner U., Forbes M.G., Willmitzer L. (2005). GC-MS libraries for the rapid identification of metabolites in complex biological samples. FEBS Lett..

[41] Ye G., Liu Y., Yin P., Zeng Z., Huang Q., Kong H., Lu X., Zhong L., Zhang Z., Xu G. (2014). Study of Induction Chemotherapy Efficacy in Oral Squamous Cell Carcinoma Using Pseudotargeted Metabolomics. J. Proteome Res..

[42] Imperlini E., Santorelli L., Orrù S., Scolamiero E., Ruoppolo M., Caterino M. (2016). Mass Spectrometry-Based Metabolomic and Proteomic Strategies in Organic Acidemias. BioMed Res. Int..

[43] Allwood J.W., Goodacre R. (2010). An introduction to liquid chromatographyâ€“mass spectrometry instrumentation applied in plant metabolomic analyses. Phytochem. Anal..

[44] Farag M.A., Huhman D., Lei Z., Sumner L. (2007). Metabolic profiling and systematic identification of flavonoids and isoflavonoids in roots and cell suspension cultures of Medicago truncatula using HPLC–UV–ESI–MS and GC–MS. Phytochemistry.

[45] Rogachev I., Aharoni A. (2011). UPLC-MS-Based Metabolite Analysis in Tomato. Adv. Struct. Saf. Stud..

[46] Doerfler H., Lyon D., Nägele T., Sun X., Fragner L., Hadaček F., Egelhofer V., Weckwerth W. (2012). Granger causality in integrated GC–MS and LC–MS metabolomics data reveals the interface of primary and secondary metabolism. Metabolomics.

[47] Han J.S., Lee S., Kim H.Y., Lee C.H. (2015). MS-Based Metabolite Profiling of Aboveground and Root Components of Zingiber mioga and Officinale. Molecules.

[48] Cuadros-Inostroza A., Ruiz-Lara S., González E., Eckardt A., Willmitzer L., Peña-Cortés H. (2016). GC–MS metabolic profiling of Cabernet Sauvignon and Merlot cultivars during grapevine berry development and network analysis reveals a stage- and cultivar-dependent connectivity of primary metabolites. Metabolomics.

[49] Moing A., Aharoni A., Biais B., Rogachev I., Meir S., Brodsky L., Allwood J.W., Erban A., Dunn W., Kay L. (2011). Extensive metabolic cross-talk in melon fruit revealed by spatial and developmental combinatorial metabolomics. New Phytol..

[50] Logemann E., Tavernaro A., Schulz W., Somssich I., Hahlbrock K. (2000). UV light selectively coinduces supply pathways from primary metabolism and flavonoid secondary product formation in parsley. Proc. Natl. Acad. Sci. USA.

[51] Liu C.-J., Deavours B.E., Richard S.B., Ferrer J.-L., Blount J.W., Huhman D., Dixon R.A., Noel J.P. (2006). Structural Basis for Dual Functionality of Isoflavonoid O-Methyltransferases in the Evolution of Plant Defense Responses[OA]. Plant Cell.

[52] Liu C.-J., Huhman D., Sumner L., Dixon R.A. (2003). Regiospecific hydroxylation of isoflavones by cytochrome p450 81E enzymes from Medicago truncatula. Plant J..

[53] Dhaubhadel S., McGarvey B.D., Williams R., Gijzen M. (2003). Isoflavonoid biosynthesis and accumulation in developing soybean seeds. Plant Mol. Boil..

[54] Graham M.Y. (2005). The Diphenylether Herbicide Lactofen Induces Cell Death and Expression of Defense-Related Genes in Soybean1. Plant Physiol..

[55] Jiao J., Gai Q.-Y., Wang W., Luo M., Gu C.-B., Fu Y.-J., Ma W. (2015). Ultraviolet Radiation-Elicited Enhancement of Isoflavonoid Accumulation, Biosynthetic Gene Expression, and Antioxidant Activity in Astragalus membranaceus Hairy Root Cultures. J. Agric. Food Chem..

[56] Caldwell M.M., Ballare C.L., Bornman J.F., Flint S.D., Bjorn L.O., Teramura A.H., Kulandaivelu G., Tevini M. (2003). Terrestrial ecosystems, increased solar ultraviolet radiation and interactions with other climatic change factors. Photochem. Photobiol. Sci..

[57] Xiong F.S., Day T.A. (2001). Effect of solar ultraviolet-B radiation during springtime ozone depletion on photosynthesis and biomass production of Antarctic vascular plants. Plant Physiol..

[58] Xu R.Y., Nan P., Yang Y., Pan H., Zhou T., Chen J. (2011). Ultraviolet irradiation induces accumulation of isoflavonoids and transcription of genes of enzymes involved in the calycosin-7-O-beta-d-glucoside pathway in Astragalus membranaceus Bge. var. mongholicus (Bge.) Hsiao. Physiol. Plant.

[59] Liu Y., Liu J., Wang Y., Abozeid A., Tian D., Zhang X.-N., Tang Z. (2017). The Different Resistance of Two Astragalus Plants to UV-B Stress is Tightly Associated with the Organ-specific Isoflavone Metabolism. Photochem. Photobiol..

[60] Li J., Ou-Lee T.M., Raba R., Last A.R.L. (1993). Arabidopsis flavonoid mutants are hypersensitive to UB-B irradiation. Plant Cell..

[61] Liu L., McClure J.W. (1995). Effects of UV-B on activities of enzymes of secondary phenolic metabolism in barley primary leaves. Physiol. Plant..

[62] Arnon D.I. (1949). Copper Enzymes in Isolated Chloroplasts. Polyphenoloxidase in Beta Vulgaris. Plant Physiol..

[63] Aiamla-Or S., Shigyo M., Ito S.-I., Yamauchi N. (2014). Involvement of chloroplast peroxidase on chlorophyll degradation in postharvest broccoli florets and its control by UV-B treatment. Food Chem..

[64] Cuadros-Inostroza Á., Caldana C., Redestig H., Kusano M., Lisec J., Peña-Cortés H., Willmitzer L., Hannah M. (2009). TargetSearch-a Bioconductor package for the efficient preprocessing of GC-MS metabolite profiling data. BMC Bioinform..

[65] Langfelder P., Horvath S. (2008). WGCNA: An R package for weighted correlation network analysis. BMC Bioinform..

[66] Dileo M.V., Strahan G.D., Bakker M.D., Hoekenga O. (2011). Weighted Correlation Network Analysis (WGCNA) Applied to the Tomato Fruit Metabolome. PLoS ONE.

[67] Liu J., Liu Y., Wang Y., Zhang Z.-H., Zu Y.-G., Efferth T., Tang Z.-H. (2016). The Combined Effects of Ethylene and MeJA on Metabolic Profiling of Phenolic Compounds in Catharanthus roseus Revealed by Metabolomics Analysis. Front. Physiol..

[68] Zu Y.-G., Pang H.-H., Yu J.-H., Li D.-W., Wei X.-X., Gao Y.-X., Tong L. (2010). Responses in the morphology, physiology and biochemistry of Taxus chinensis var. mairei grown under supplementary UV-B radiation. J. Photochem. Photobiol. B Boil..

[69] Soriano-Melgar L.D.A.A., Alcaraz-Meléndez L., Méndez-Rodríguez L.C., Puente M.E., Rivera-Cabrera F., Zenteno-Savin T. (2014). Antioxidant responses of damiana (Turnera diffusa Willd) to exposure to artificial ultraviolet (UV) radiation in an in vitro model; part ii; UV-B radiation. Nutr. Hosp..

[70] Dias D.A., Hill C.B., Jayasinghe N.S., Atieno J., Sutton T., Roessner U. (2015). Quantitative profiling of polar primary metabolites of two chickpea cultivars with contrasting responses to salinity. J. Chromatogr. B.

[71] Harborne J.B., A Williams C. (2000). Advances in flavonoid research since 1992. Phytochemistry.

[72] Peer W., Murphy A.S. (2007). Flavonoids and auxin transport: Modulators or regulators?. Trends Plant Sci..

[73] Taylor L.P., Grotewold E. (2005). Flavonoids as developmental regulators. Curr. Opin. Plant Boil..

[74] Ryan K.G., Swinny E.E., Markham K.R., Winefield C. (2002). Flavonoid gene expression and UV photoprotection in transgenic and mutant Petunia leaves. Phytochemistry.

[75] Ryan K.G., Swinny E.E., Winefield C., Markham K.R. (2001). Flavonoids and UV photoprotection in Arabidopsis mutants. Z. Nat. C.

[76] Gibon Y., Pyl E.-T., Sulpice R., Lunn J., Höhne M., Günther M., Stitt M. (2009). Adjustment of growth, starch turnover, protein content and central metabolism to a decrease of the carbon supply when Arabidopsisis grown in very short photoperiods. Plant Cell Environ..

[77] Robbins M.P. (2000). Biochemistry of plant secondary metabolism. Annual Plant Reviews, Volume 2. Edited by Michael Wink. Eur. J. Plant Pathol..

[78] Kusano M., Fukushima A., Redestig H., Saito K. (2011). Metabolomic approaches toward understanding nitrogen metabolism in plants. J. Exp. Bot..

[79] Agati G., Cerovic Z.G., Pinelli P., Tattini M. (2011). Light-induced accumulation of ortho-dihydroxylated flavonoids as non-destructively monitored by chlorophyll fluorescence excitation techniques. Environ. Exp. Bot..

[80] Zhang Z.-Z., Che X.-N., Pan Q.-H., Li X.-X., Duan C.-Q. (2013). Transcriptional activation of flavan-3-ols biosynthesis in grape berries by UV irradiation depending on developmental stage. Plant Sci..

[81] Kanazawa K., Hashimoto T., Yoshida S., Sungwon P., Fukuda S. (2012). Short Photoirradiation Induces Flavonoid Synthesis and Increases Its Production in Postharvest Vegetables. J. Agric. Food Chem..

[82] Singh K., Kumar S., Rani A., Gulati A., Ahuja P.S. (2008). Phenylalanine ammonia-lyase (*PAL*) and cinnamate 4-hydroxylase (*C4H*) and catechins (flavan-3-ols) accumulation in tea. Funct. Integr. Genom..

